# Thrombin generation in low plasma volumes

**DOI:** 10.1186/s12959-018-0164-6

**Published:** 2018-05-15

**Authors:** Saartje Bloemen, Hilde Kelchtermans, H. Coenraad Hemker

**Affiliations:** 1 Synapse Research Institute, Cardiovascular Research Institute Maastricht, Maastricht University Medical Center, Maastricht, the Netherlands; 2 Department of Biochemistry, Cardiovascular Research Institute Maastricht, Maastricht University Medical Center, Maastricht, the Netherlands

**Keywords:** Thrombin generation, Calibrated automated thrombinography, Low volumes

## Abstract

Accurate thrombin generation determination by calibrated automated thrombinography can be sustained when reducing the plasma and reagent volumes up to half, but not for higher reductions or plasma dilutions.

Calibrated automated thrombinography (CAT) i.e. the measurement of the complete course of the thrombin concentration (thrombin generation, TG) in clotting blood(−plasma), is a global function test of the coagulation system. Whereas clotting assays measure only the initiation phase of TG, the full TG curve provides information on the propagation and decay phase as well and therefore also reflects the inhibitory factors (tissue factor pathway inhibitor, proteins C and S and antithrombin). Since sample volumes can be limited in case of e.g. pre-existing sample sets or in pediatric studies, we attempted to minimize the volume of plasma necessary to obtain a TG curve with CAT and found that for volumes below 40 μl of plasma (i.e. final volume of 60 μl) the experimental precision cannot be maintained.

Previous studies showed that the total amount of thrombin activity (endogenous thrombin potential, ETP) as represented by the area under the TG curve, as well as the peak thrombin activity have been shown to correlate significantly with the risk of bleeding as well as venous thrombosis [1–10]. A relation with arterial thrombosis does exist, but is less straightforward [11, 12]. In TG measurements, adequate calibration requires compensation for substrate consumption and inner filter effect. Therefore, a calibrator must be run in parallel with the sample in which TG is measured, which doubles the amount of plasma required; moreover, the experiments are preferably executed in duplicate. Also, if maximal information on the TG system is to be obtained, experiments in the presence of different concentrations of tissue factor (TF), with and without added thrombomodulin (TM) or activated protein C should be performed. Thus, although the volume necessary per CAT measurement (< 500 μl) is comparable to that of other clotting assays, it is a question of significant practical importance whether the volume of plasma required per test can be reduced.

Earlier results show that diminishing the plasma volume without decreasing the total volume, i.e. diluting the plasma further than 2:3, seriously impairs the sensitivity of the method to factors other than pro- and antithrombin [13]. We therefore opted for decreasing the volume of all reactants.

First, we evaluated different reductions of the final volume (90 μl, 60 μl and 30 μl). At volumes < 1/2 (i.e. < 60 μl) of the original the experimental error increased significantly (data not shown), so we decided to further develop a method in which all volumes are halved, the ‘MidiCAT’.

In a MidiCAT measurement, 10 μl of trigger and 40 μl of platelet poor plasma (PPP) are added in a well of a round-bottom 96-well plate (Immulon 2HB) and after 5 min incubation the reaction is started by dispensing 10 μl of substrate-recalcification mixture. The plasma ratio as well as the final concentrations are the same as in the CAT method, i.e. 5 pM TF (Innovin, Dade-Behring, Marburg, Germany), 4 μM phospholipids (PL) (PS/PE/PC 20/20/60%, Avanti Polar Lipids Inc., Alabaster, AL, USA) in Hepes buffer containing 5 mg/ml bovine serum albumin in addition to 416.7 μM Z-Gly-Gly-Arg-aminomethylcoumarin (Bachem) and 16.7 mM CaCl_2_ in Hepes buffer containing 60 mg/ml bovine serum albumin (FluCa). The same TF/PL solution was used for analysis of one set of samples with both measurement procedures. FluCa was freshly prepared for each run using the same batch of reagents for both procedures. For each set of samples both procedures were performed on the same day. Normal pooled plasma (NPP) was included in each run (*n* = 4). Forty-three samples were tested with both procedures. According to the Clinical and Laboratory Standards Institute (CLSI) guidelines for method procedure comparison when introducing a different measurement procedure, at least 40 patient samples should be measured that span the measuring interval of the assay. Samples were chosen to cover the range for the ETP and peak, since these are the most important variables in CAT. All healthy donors (*n* = 37) and patients (*n* = 6) provided informed consent and the study protocol was evaluated by the local medical ethical committee. Donors were considered healthy when they were not using any anticoagulant drugs and had no history of bleeding or thrombosis. Donors using oral contraceptives with known high ETP and peak values were chosen to cover the higher range of values. Patients using vitamin K antagonists were included to provide samples in the lower range of the ETP and peak values. Plasmas from two healthy donors and two different NPP were also analyzed in the absence of TF, using both measurement procedures, to evaluate the level of contact activation.

Spearman correlation coefficients were defined to assess the correlation between the two methods. Bland-Altman plots were constructed to evaluate the differences and bias in results between the two procedures. Here the percentage difference of the two methods (100*(A-B)/average) versus the average ((A + B)/2) of the two methods was analyzed (method A: MidiCAT, method B: regular CAT). Passing-Bablok regression was performed to analyze the comparability of the measurements. Analyses were performed using GraphPad Prism (version 5.00) or MedCalc (version 17.7.2).

We compared TG in PPP of 43 healthy donors using the original CAT and our MidiCAT tested in duplicate. The correlation between the two techniques was very good for all parameters (Fig. 1, left panels; Spearman correlation coefficients for all parameters above 0.940, apart from lag time: r_s_ = 0.832). The intra-assay variation for the new assay ranged between 0.5 and 2.0% for the four TG parameters. The inter-assay CV was for lag time: 11.0%, ETP: 6.5%, peak: 4.7% and time-to-peak: 5.4% for the regular CAT (determined in NPP in 37 runs) and respectively 8.6%, 3.6%, 3.1% and 5.4% for the MidiCAT (determined in NPP in 34 runs). The medians and interquartile ranges are shown in Table 1. The bias as deducted from the Bland-Altman curves was between − 0.93% and 5.3% (Fig. 1, right panels). At lower ETP and peak levels the regular CAT resulted in higher values and at higher ETP and peak levels the MidiCAT resulted in higher values. For the time-dependent parameters the distribution was more even. Passing-Bablok regression equations were for ETP y = 1.14 (95% CI: 1.05 to 1.27)x - 124.086 (95% CI: -260.51 to − 45.05), for peak y = 1.09 (95% CI: 1.03 to 1.17)x – 9.231 (95% CI: -22.61 to 2.36), for lag time y = 1.00 (95% CI: 0.99 to 1.01)x + 0.00 (95% CI: -0.01 to 0.01) and for time-to-peak y = 0.99 (95% CI: 0.92 to 1.00)x + 0.011 (95% CI: 0.00 to 0.22). These equations indicate similarity between the two procedures for all four parameters. Additionally, the level of contact activation between the two measurement procedures was estimated (at 0 pM TF and 4 μM PL), providing similar results (data not shown).

**Fig. 1:**
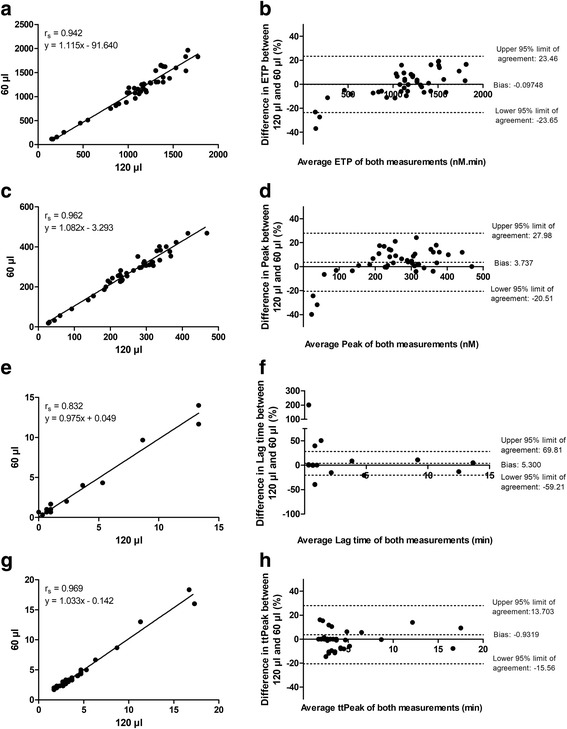
Correlation and Bland-Altman plots of thrombin generation parameters in MidiCAT and regular CAT. The correlation between the MidiCAT (60 μl final volume) and the regular CAT (120 μl final volume) was determined for the four main CAT parameters i.e. **a** endogenous thrombin potential (ETP), (**c**) peak, (**e**) lag time and (**g**) time-to-peak (ttPeak) in 43 healthy donors using 5 pM tissue factor (measured in duplicate). The Spearman correlation coefficient is indicated. Bland-Altman plots were constructed analyzing the percentage difference of the two methods (100*(A-B)/average) versus the average ((A + B)/2) of the two methods. Method A was the MidiCAT and method B was the regular CAT. Plots were prepared for (**b**) ETP, (**d**) peak, (**f**) lag time and (**h**) ttPeak. For all analyses 43 subjects were measured. Of note, for the lag time and time-to-peak several subjects had the same result, because of which it seems that less dots are depicted in the graphs

**Table 1:** Median and interquartile ranges of MidiCAT (60 μl) and regular CAT (120 μl) in 43 subjects

		25% Percentile	Median	75% Percentile
Lag time (min)	120 μl	0.67	0.67	1.00
	60 μl	0.67	0.67	1.00
ETP (nM.min)	120 μl	988.8	1169.0	1362.0
	60 μl	962.0	1174.0	1401.0
Peak (nM)	120 μl	198.7	264.1	319.4
	60 μl	226.7	282.5	333.6
Time-to-peak (min)	120 μl	2.67	3.00	4.30
	60 μl	2.67	3.00	4.00

We conclude that accurate measurement of TG curves is feasible in half the volume of the original method, but not less. Since there is a higher surface-to-volume ratio, it could be postulated that there may potentially be a higher level of contact activation in the MidiCAT. However, we found similar results between both procedures. Looking at the Bland-Altman curves, the bias was higher at the lower end of the measurement range. Our measurements were performed with in-house reagents using 5 pM as a final TF concentration, which are comparable to the commercially available reagents for CAT (Stago PPP-Reagent®). However, for other TF concentrations the differences are not yet known. Additionally, although similar results are obtained with both measurement procedures, we recommend using one procedure within one study.

## Abbreviations

CAT: calibrated automated thrombinography
CLSI: Clinical and Laboratory Standards Institute
ETP: endogenous thrombin potential
NPP: normal pooled plasma
PL: phospholipids
PPP: platelet poor plasma
TF: tissue factor
TG: thrombin generation
TM: thrombomodulin
